# Cognitive Reappraisal and the Association Between Perceived Stress and Anxiety Symptoms in COVID-19 Isolated People

**DOI:** 10.3389/fpsyt.2020.00858

**Published:** 2020-09-02

**Authors:** Chen Xu, Yanjun Xu, Song Xu, Qianhui Zhang, Xiaotong Liu, Yifan Shao, Xiaoxiao Xu, Li Peng, Min Li

**Affiliations:** ^1^ Department of Military Psychology, Faculty of Medical Psychology, Army Medical University, Chongqing, China; ^2^ Department of Psychology, The 991^th^ Hospital of the Chinese PLA, Xiangyang, China

**Keywords:** perceived stress, emotion regulation, cognitive reappraisal, anxiety symptoms, COVID-19 isolated people

## Abstract

The purpose of this study is to examine how the emotion regulation strategy, cognitive reappraisal, affects the association between perceived stress and anxiety symptoms in COVID-19 isolated people. Data for this cross-sectional study come from a community-based online survey of COVID-19 isolated people (*N* = 328), who are not infected with the 2019-nCoV virus. We applied correlation and moderating effect for data analysis and found that cognitive reappraisal negatively moderated the relationship between perceived stress and anxiety symptoms. These results give us a new perspective on understanding the relationship between anxiety symptoms and perceived stress by clarifying the protective function of cognitive reappraisal. It buffers the induced negative emotion when COVID-19 isolated people perceive overpressure, and thus instigates future research into targeted clinical interventions, which aim to cultivate cognitive reappraisal skills for those isolated people in the face of stressful events or crisis events.

## Introduction

At the end of 2019, a new type of coronavirus (COVID-19) came quietly, invading Wuhan and Hubei in a short time, and soon spreading to the whole country. In order to control the further spread of the virus and cut off the route of infection, besides the medical treatment of the virus-infected patients, medical isolation is also conducted for those in close contact with the infected patients. The indirect contacted will be isolated at home. Isolated people have read various news about the epidemic through the online news media. Faced with the invisible deadly virus, isolated people are afraid of being infected, and worried about the health of their relatives and friends. They usually face the dual pressures of infection and lack of social links during isolation. All of these has become stressors for the isolated people, eliciting their stress reactions. Existing studies have found that perceived stress can reduce immunity (1, 2) and increase anxiety symptoms (3), while the reduction in immunity increases the risk of infection and eventually complicates the prevention and control of the epidemic. Although measures are taken to alleviate the physical problems of people in quarantine, mental health receives little attention (4).

Stress can be thought of as the strain that coexists with demand (5), which can trigger series of hormonal, neurological, and other biological “fight or flight” responses. Perceived stress is a subjective response to a threat in any given situation, generally a short-term experience (5). Anxiety symptoms is the anticipation of unpredictable, impending threats or stress (6). It is a persistent psychological disorder that does not disappear with the threat. Besides the feelings of fear and panic, excessive and persistent anxiety also causes a series of physiological symptoms, such as fatigue, headache, sweating, abdominal pain, etc. (6). After an individual has suffered a stressful event, they will have a judgment on the severity of the crisis in terms of perception and cognition, so that there will be a certain degree of reaction in physiological and psychological degrees. Anxiety reaction is one of them. The more stress they perceived, the more anxious they are. If the stress cannot be resolved for a long time, the anxiety will change into an anxiety state for a certain period of time and reach the relevant anxiety symptom standard. A lot of researches have shown the close relationship between anxiety and perceived stress, and both acute and chronic stress exposure could lead to anxiety disorders (7–9).

However, individuals’ responses to stress are quite heterogeneous (10). Facing the same stress event, individuals can perceive it as either challenge or threat, which will lead to either positive or negative results (5). Stress can be the motivation for greater achievements if you take it as a challenge (11). Conversely, viewing stress as a threat may cripple individuals into ineffectiveness and even cause severe psychological disorders such as anxiety and depression. Therefore, adopting different coping strategies and emotional regulation strategies in the face of stress may bring different emotional experiences (12). Failing to effectively manage one’s emotional response to daily life events will lead to longer and more severe periods of pain, and that may eventually develop into anxiety and depression disorder (13). In fact, research on the role of emotion regulation in the development and treatment of psychopathology has been increasing in recent years, and the importance of emotion regulation in adjustment to stress has been emphasized (14, 15). According to Gross and Munoz (16), emotion regulation is involved in anxiety crucially, it may be an important protective factor against increasing in anxiety symptoms that ones are able to use adaptive emotion regulation strategies.

Emotion regulation strategies include cognitive and behavioral strategies and acceptance strategies, which can change the intensity and duration of emotion (17). It is important to understand what kind of emotion regulation strategies contribute to effective emotion regulation, because there are many different strategies in regulating their emotions (18). Cognitive appraisal refers to the individual’s estimation of the nature, degree and possible harm of the stressor encountered from his own point of view, and also estimates the resources of the stressor that the individual can use when facing the stressor (19).

Cognitive reappraisal is a type of emotion regulation strategies proposed by Gross. Cognitive reappraisal is an individual’s intention to selectively interpret the meaning of an event, it directly aims at appraisals by changing the subjective evaluation of an emotion producing situation (20). Cognitive reappraisal is useful because it helps us to recognize the time when our thoughts have fallen into a negative mode and allows us to down regulate negative emotions in time and turn them into positive ones (21). It has been showed that cognitive reappraisal is associated with less depression, fewer negative effects, and higher life satisfaction (22, 23). Previous studies compared the use of emotion regulation strategies in healthy controls and those with clinical level of anxiety and/or depression and found that the former uses cognitive reappraisal significantly more frequently (24, 25). Thus, cognitive reappraisal is a protective factor for psychopathology.

According to the appraisal theories of emotion, it is an individual’s subjective appraisal of an event that leads to a specific emotional reaction rather than the event itself (26). Previous study has found that the initial negative cognition and beliefs that people generate after trauma are related to the depression, anxiety, and PTSD (27). It is thought to be a key ingredient of many psychological interventions such as cognitive (28) and cognitive-behavioral therapy (CBT) (16) that learning to change the appraisals in emotional situations. Therefore, if the individual’s cognition can be transformed when facing the stressful life events, the individual’s subsequent emotional experience can be achieved naturally. Cognitive reappraisal helps people view stress as a positive challenge rather than a negative threat, so that they can manage stress better. Researchers have found that consistent use of cognitive reappraisal is associated with increased positive affect, decreased negative affect, as well as better interpersonal functioning (20, 29, 30). Individuals with anxiety symptoms have been found with less frequent and effective use of cognitive reappraisal (31, 32). On the contrary, maladaptive emotion appraisal skills increase the difficulties to recover from negative mood following a stressful event (33). Specifically, the benefits of cognitive reappraisal use have been highlighted by studies and raise the question of whether this strategy is useful in the context of overpressure from isolation of COVID-19 as well. Overall, these studies suggested that cognitive reappraisal had a buffering effect on the relationship between stress and negative outcomes, including anxiety symptoms. Specifically, cognitive reappraisal may function as a vital protective factor against anxiety symptoms by providing an effective way to down-regulate negative emotions.

Based on the above concerns, we hypothesize that perceived stress is positively associated with anxiety symptoms, cognitive reappraisal will attenuate the association between perceived stress and anxiety symptoms in COVID-19 isolated people. This model can help us to understand the risk factors of anxiety symptoms and guide the prevention and intervention plan of high-risk population. We hope to help isolated people better deal with crisis events in daily life, reduce the risk of psychological problems, and maintain mental health.

## Materials and Methods

### Participants and Procedure

Prior to the study, it had been approved by the Ethics Committee of Army Medical University of China. Between Feb. 8^th^ and Feb. 21^st^, we conducted a cross-sectional online survey in mainland China. The researchers posted this survey on the Internet. Participants are recruited online, and all are willing to fill out the self-administered questionnaires. A convenience sample of 328 isolated people completed the questionnaire. During the investigation, all participants were quarantined and were not infected with the 2019-nCoV virus.

The mean age of the participants was 30.96 (*SD* = 6.84) years, in a range of 18 to 56 years. Distribution of the sample by gender was 60.98% (*n* = 200) male and 39.02% (*n* = 128) female.

### Measures

#### Demographic Information

We collected demographic information of participants including age, gender, educational background, and marital status. **Table 1** shows the demographic characteristics of the sample.

**Table 1:** Demographic data of the sample.

	N (%)	M (SD) Range
Age		30.96 (6.84) 18-56
Gender		
Female	128 (39.02)	
Male	200 (60.98)	
Marital status		
Married	203 (60.89)	
Unmarried	125 (38.11)	
Educational background		
Junior high school and below	40 (12.19)	
High school	65 (19.82)	
University or collage	205 (65.50)	
Graduate and above	18 (5.49)	

M, mean; SD, standard deviation.

#### Perceived Stress

Perceived Stress Scale (PSS) (34) served as an assessment tool to measure the subjective pressure that individuals perceive in the past month, which is available in PSS-14, PSS-10, and PSS-4 versions. This study selected the Chinese version of PSS-14 (CPSS-14) (35), including 7 negative description items and 7 positive feeling description items. There are typical items like “have you been upset by something that happened unexpectedly?” and “have you felt that things were going your way?” Each item is using a 5-point Likert scale (1 = “never”; 5 = “always”). The scores of 14 items are added to get the total score. The higher the total score, the more stress the individual perceives in the past month. The CPSS-14 has showed high internal consistency and test-reliability.

#### Cognitive Reappraisal

Cognitive reappraisal was measured based on the Emotion Regulation Questionnaire (ERQ) (20). ERQ is a 10-item self-report measurement scale, including two subscales: cognitive reappraisal and expression inhibition, which is used to measure individual’s expression inhibition and cognitive reappraisal. In this study, only cognitive reappraisal subscale was used, including 6 items. The sample items include “When I want to feel happier, I think about something different” and “I control my feelings about things by changing the way I think about them”. Each item used a 7-point Likert scale (1 = strongly disagree; 7 = strongly agree). The scores of all items are added together, the higher the total score is, the more frequently the cognitive appraisal strategy is used. The ERQ has showed high test-reliability and internal consistency (20), and the Cronbach’s alpha values for the Chinese version of ERQ are acceptable (36).

#### Anxiety

We used Zung’s Self-Rating Anxiety Scale (SAS) (37) to assess the subjective feelings of individuals with symptoms of anxiety in the past week. The main assessment basis of SAS is the frequency of symptoms defined by the items divided into 4 levels: from “never” to “always”. The SAS includes 20 items, with typical items like “Do you feel more nervous and anxious than usual?” and “Are you bothered by headaches, neck and back pains?” The scores of all items are added and converted to obtain standard scores. The higher the score, the more anxious. The Chinese translation of the survey has been previously validated (38).

#### Power Analysis

Since we adopted a convenient sampling method to obtain 328 samples, in the interaction analysis model, the effect size *f²* = 0.04, *α* = 0.05. As a consequence, the observed power 1-*β* = 0.89.

### Analyses

IBM SPSS 24.0 statistical software was used for descriptive data, correlation, and regression analysis. We used Ordinary Least Squares (OLS) to test our research hypothesis. Firstly, we studied the relationship between perceived stress and anxiety symptoms (model 1), and the relationship between cognitive reappraisal and anxiety symptoms (model 2). Secondly, we studied the main effects of perceived stress and cognitive reappraisal on anxiety symptoms (model 3). Finally, we studied the interaction of cognitive reappraisal and perceived stress on anxiety symptoms (model 4). To fully examine important interactions, we followed the guidelines provided by Preacher, Curran, and Bauer (39). All continuous variables were standard, and all categorical variables were coded for effect (1.0 and 2.0).

In this study, we set anonymous responses and reverse scores to control the possible common method bias, and the common method bias was tested using the Herman single factor method (40). Exploratory factor analysis showed that a total of eight factors were obtained, and the interpretation rate of the first factor was 25.60%, which was <40% of the reference value. This indicated there is no serious common method bias.

## Results

### Description Statistics

See **Table 2** for descriptive results. The α reliability of the perceived stress scale was 0.841 (*M* = 33.54, *SD* = 7.44), that of the cognitive reappraisal scale was 0.872 (*M* = 32.95, *SD* = 7.11), and that of the anxiety symptoms was 0.844 (*M* = 28.52, *SD* = 6.74). The correlation analysis shows that among the COVID-19 isolated people, there are moderate relationships between the variables. Specifically, perceived stress was significantly positively correlated with anxiety symptoms (*r* = 0.61, *p* < 0.001), but negatively correlated with cognitive reappraisal (*r* = -0.34, *p* < 0.001). Moreover, there was negative correlation between cognitive reappraisal and anxiety symptoms (*r* = -0.46, *p* < 0.001).

**Table 2:** Intercorrelations and descriptive statistics between variables.

Variables	M	SD	1	2	3
1.Perceived stress	33.54	7.44	(0.841)		
2.Cognitive reappraisal	32.95	7.11	-0.34^***^	(0.872)	
3.Anxiety symptoms	28.52	6.74	0.61^***^	-0.46^***^	(0.844)

α coefficient in diagonal line. ***p < 0.001.

### Moderation Analysis

**Table 3** displays the OLS regression models applied to test our research hypotheses. We separately examined the main effects of perceived stress (Model 1) and the emotion regulation skill cognitive reappraisal (Model 2) on anxiety symptoms. The results showed that the high level of perceived stress was associated with the high level of anxiety symptoms (*β* = 0.62, P < 0.001); the high level of cognitive reevaluation was associated with the low level of anxiety symptoms (*β* = 0.47, P < 0.001). Perceived stress was connected with higher levels of anxiety symptoms (*β* = 0.62, *p* < 0.001); higher levels of the cognitive reappraisal were associated with lower levels of anxiety symptoms (*β* = -0.47, *p* < 0.001). Then, in model 3, we examined the main effects of cognitive reappraisal and perceived stress on anxiety symptoms. The results showed that the effects of perceived stress (*β* = 0.52, *p* < 0.001) and cognitive reappraisal (*β* = -0.28, *p* < 0.001) decreased slightly, but both main effects were still statistically significant. To test the moderation hypothesis, we then investigated the potential moderating effect of cognitive reappraisal on the relationship between perceived stress and anxiety symptoms. As seen in **Table 3**, the interaction of moderation is significant (*β* = -1.34, *p* < 0.001, Model 4). For the increase of the interaction of the model, *R²* change significantly, *R²* change = 0.02, *F*1,320) = 12.32, *p* < 0.001.

**Table 3:** OLS regression models examining associations of perceived stress and emotion regulation on depressive symptoms.

Variables	Model 1		Model 2		Model 3		Model 4	
	β	t	β	t	β	t	β	t
Gender	-0.11	-2.39^*^	0.03	0.62	-0.06	-1.41	-0.05	-1.23
Age	0.05	1.01	-0.04	-0.77	0.03	0.71	0.03	0.71
Perceived stress	0.62	13.95^***^			0.52	11.51^***^	0.52	11.77^***^
Cognitive reappraisal			-0.47	-9.42^***^	-0.28	-6.29^***^	-0.24	-5.28^***^
Perceived stress X cognitive reappraisal							-0.13	-3.51^**^
R^2^	0.38		0.22		0.45		0.47	
F	65.17^***^		29.86^***^		64.62^***^		55.98^***^	

All models control for age and gender; each variable in the model is standardized. *p < 0.05,**p < 0.01,***p < 0.001.

**Figure 1:**
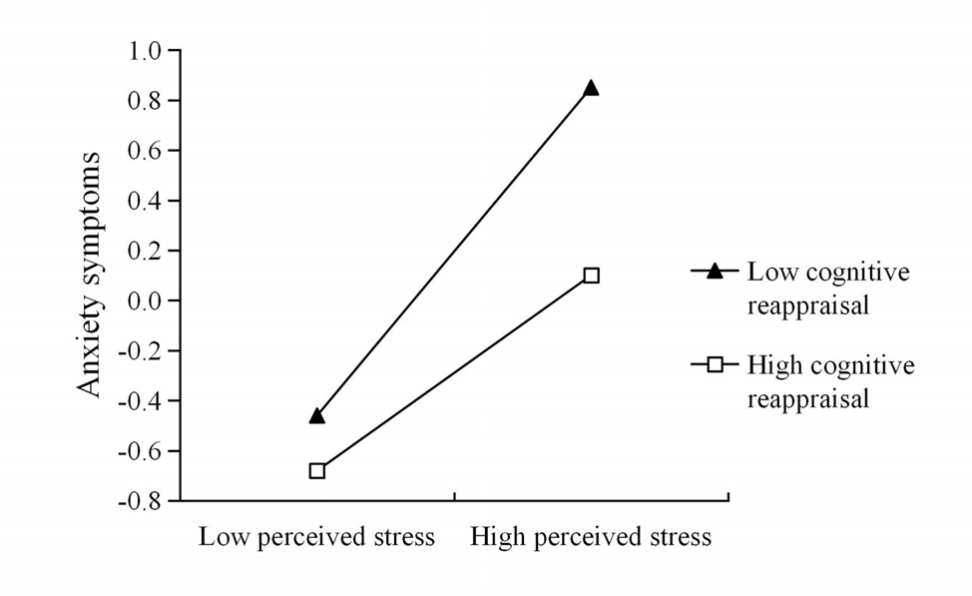
Cognitive reappraisal moderates the relation between perceived stress and anxiety symptoms.

In order to test the interaction, according to the procedure outlined by Aiken and West (41), the *M* ± 1SD of cognitive reappraisal and perceived stress was used to plot the relationship. Simple slopes analysis showed that perceived stress had a significant positive predictive effect on anxiety symptoms in individuals with low cognitive reappraisal (*M* − 1*SD*), simple slope = 0.66, *t* = 11.09, *p* < 0.001; For the subjects with high cognitive reappraisal (*M* + 1*SD*), there is still a significant positive predictive effect between perceived stress and anxiety symptoms, but the predictive power is lower, simple slope = 0.39, *t* = 6.74, *p* < 0.001, suggesting at high levels of cognitive reappraisal, participants with high perceive stress had lower levels of anxiety symptoms than those with low cognitive reappraisal (see **Figure 1**). That is, the positive consequences of perceived stress on anxiety symptoms appeared to be greater for those with lower cognitive reappraisal compared with those with higher levels of cognitive reappraisal. Thus, for those isolated people with perceived stress, the cognitive reappraisal provides an internal resource to reduce anxiety.

## Discussion

On average, perceived stress leads to increases in a wide range of negative psychological outcomes, including anxiety symptoms (42, 43). Our study, however, focuses on the observation that some people do not experience elevated anxiety symptoms. Stress caused by the COVID-19 outbreak is often inherently emotional, so the best way to adjust is to change cognition. Cognitive reappraisal refers to changing the understanding of emotional events and the understanding of the personal significance of emotional events (20). When adopting cognitive reappraisal strategies, individuals will look at stressful life events from different perspectives, thus avoiding being trapped in negative emotions. Therefore, cognitive reappraisal is an effective measure of coping with stress (44) and a protective factor of anxiety symptoms (45, 46). Our study tests the hypothesis that cognitive reappraisal moderates the relationship between perceived stress and anxiety symptoms in COVID-19 isolated people.

Firstly, we explored the relationship between perceived stress, cognitive reappraisal, and anxiety symptoms among COVID-19 isolated people. Our study replicated the frequently reported positive relationship between perceived stress and anxiety symptoms (47–49). Secondly, we found that cognitive reappraisal was negatively correlated with anxiety symptoms, and negatively correlated with perceived stress. Thirdly, the hypothesized interaction between cognitive reappraisal and perceived stress in predicting anxiety symptoms was found. More concretely, for the COVID-19 isolated people with higher cognitive reappraisal ability, the relationship between perceived stress and anxiety symptoms is weaker. Therefore, cognitive reappraisal buffered the occurrence of anxiety symptoms, when COVID-19 isolated people perceived overpressure. Consistent with our finding in this study, other research has found similar association between perceived stress and anxiety symptoms (7–9). Due to lack of social expression and physical activities, COVID-19 isolated people who perceived stress may have increasing difficulty regulating emotions. Furthermore, the length of quarantine time is associated with increasing risk for anxiety and depression symptoms. Such stressors are linked with increasing perceived stress that may in-turn significantly influences anxiety symptoms.

Researchers have found that isolated people who perceived stress, compared with those who do not, have challenges developing anxiety symptoms (50). We found that individuals with higher perceived stress levels are more likely to have anxiety symptoms and cognitive reappraisal may buffer this impact. Our study demonstrates that perceived stress and cognitive reappraisal act collectively to influence anxiety symptoms, whereas others have found cognitive appraisal has been shown to determine the impact of stress response (51). Cognitive appraisal refers to the estimation of the stress event itself, while cognitive reappraisal is an individual’s selective interpretation of the meaning of the stress event (52). In a particular event, the individual’s interpretation of the selected meaning of the event will significantly affect the individual’s psychological experience, behavioral expression, and physiological response. Researchers have speculated that cognitive deficits may play a role in the problematic behaviors exhibited by individuals who perceived stress (53). Results of this study suggest that we should improve the cognitive reappraisal skill in COVID-19 isolated people to lessen the impact of perceived stress on anxiety symptoms. It is beneficial to target cognitive skills to reduce anxiety. Investigating the extent to which increasing cognitive reappraisal in COVID-19 isolated people decreases anxiety symptoms, would deepen understanding of the mechanisms how perceived stress raises incidence of anxiety in COVID-19 isolated people.

Our findings of the positive influence of perceived stress on anxiety symptoms have implications for COVID-19 isolated people. Individuals who are isolated, especially those who lack social connections, have significantly negative experience (54, 55). There is a growing body of evidence show that anxiety impacts on the quality of life, in a long-term state of anxiety, it is easy to produce decadent emotions and thoughts, lose interest and enthusiasm in the originally happy life, even can decrease immunity (2, 3). Thus, learning to cope with stress is critical to maintaining physical and mental health and overall well-being of isolated people under excessive pressure. Consistent with the increasing literature (47, 56, 57), our study highlights the importance of addressing overpressure and reducing anxiety symptoms in isolated people. However, the anxiety response to stress events has important adaptive impact, which helps to better mobilize our physical and mental resources, thereby improving the body’s ability to cope with environmental stress or challenges. For example, anxiety reminds us to pay more attention to the epidemic and prompts us to implement effective self-protection. These attention and active response will reduce the possibility of individuals being injured by the epidemic. Due to the threat of 2019-nCoV virus and extreme lack of social support in isolated people (4), it is not possible for them to effectively solve the overpressure. Our research highlights the importance of addressing the overpressure and alleviating anxiety symptoms of isolated people. In order to achieve these goals, we should understand the mechanism behind these connections. In general, COVID-19 isolated people tend to report higher levels of stress and anxiety. This may be due to an overall decrease in some emotion regulation skills as isolation time increases. Nonetheless, when experiencing intense emotional arousal and reducing negative reactions to stressors, COVID-19 isolated people may have more difficultly to restore homeostasis than the average person. Interventions that improve cognitive reappraisal skills will change the cognition of the isolated people, so as to reduce the harmful effects of stress and the occurrence of anxiety symptoms (43, 58). This carries a number of both theoretical and practical implications.

At present, psychological education is the main measure to reduce the pressure of COVID-19 isolated people. For stress vulnerable COVID-19 isolated people, cognitive reappraisal may have an impact on alleviating anxiety symptoms. Interventions that indirectly or directly focus on improving cognitive reappraisal skills, such as rational emotional therapy, are expected to treat overpressure in the COVID-19 isolated people. Cognitive behavioral therapy (59) and dialectical behavioral therapy (60) can also effectively develop cognitive reappraisal skills, thereby alleviating anxiety. However, in the COVID-19 isolated people, whether these therapies are effective or not still needs further study. Anxiety may have tremendous consequence on the physical and mental health of isolated people, future research in this area will be of great significance.

Several limitations of the current research are noted. First of all, the current study relies on relevant data, the cross-sectional nature of the current data limits our interpretation of the results. We cannot draw a conclusion that perceived stress have direct effect on anxiety symptoms. Eventually, stress factors appear involved in both the pathogenesis and the consequences of anxiety symptoms. Future researches should also consider that associations may vary across cultures and economic status. Secondly, we only describe the stress and anxiety status, no further intervention, future research can carry out intervention or longitudinal research to verify the effectiveness of cognitive reappraisal. Finally, although we attempted to control some of these variables, it is also the case that the covariates may influence pathways in an unknown manner, affecting the precision of results or even generating biasing results.

## Conclusion

Stress is often associated with anxiety symptoms, which may be especially detrimental to health and well-being of COVID-19 isolated people. Cognitive reappraisal skill is an important factor in determining the extent to which an individual’s perceived stress affects anxiety symptoms. In our cross-sectional sample of COVID-19 isolated people, the association between perceived stress and anxiety symptoms is influenced by the cognitive reappraisal. The positive influence of perceived stress on anxiety symptoms is greater for those with lower levels of cognitive reappraisal compared with those who have higher levels of cognitive reappraisal. The positive relationship between perceived stress and anxiety symptoms is of adaptive impact. The reinforcement of cognitive reappraisal skills has benefits for COVID-19 isolated people, particularly those vulnerable to stress, and will reduce the incidence of anxiety symptoms.

## Data Availability Statement

The raw data supporting the conclusions of this article will be made available by the authors, without undue reservation.

## Ethics Statement

The studies involving human participants were reviewed and approved by Ethics Committee of Army Medical University of China. Written informed consent for participation was not required for this study in accordance with the national legislation and the institutional requirements.

## Author Contributions

CX: Writing Original Draft, Investigation, Methodology, Software, Conceptualization. YX: Investigation, Methodology, Software, Writing and Review. SX: Resources, Editing and Review. QZ: Writing, Editing and Review. YS: Resources, Writing. XL: Resources, Review. XX: Resources, Writing. PL: Investigation, Editing and Review. ML: Supervision, Investigation, Review, Editing.

## Conflict of Interest

The authors declare that the research was conducted in the absence of any commercial or financial relationships that could be construed as a potential conflict of interest.
